# Introduction to Infrared and Raman-Based Biomedical Molecular Imaging and Comparison with Other Modalities

**DOI:** 10.3390/molecules25235547

**Published:** 2020-11-26

**Authors:** Carlos F. G. C. Geraldes

**Affiliations:** 1 Department of Life Sciences, Faculty of Science and Technology, University of Coimbra, Calçada Martim de Freitas, 3000-393 Coimbra, Portugal; geraldes@ci.uc.pt; Tel.: +351-96-766-1211; 2 Chemistry Center, Rua Larga, University of Coimbra, 3004-535 Coimbra, Portugal; 3 CIBIT-Coimbra Institute for Biomedical Imaging and Translational Research, Azinhaga de Santa Comba, 3000-548 Coimbra, Portugal

**Keywords:** bio-imaging, vibrational spectroscopy, infrared, Raman, multimodal imaging

## Abstract

Molecular imaging has rapidly developed to answer the need of image contrast in medical diagnostic imaging to go beyond morphological information to include functional differences in imaged tissues at the cellular and molecular levels. Vibrational (infrared (IR) and Raman) imaging has rapidly emerged among the molecular imaging modalities available, due to its label-free combination of high spatial resolution with chemical specificity. This article presents the physical basis of vibrational spectroscopy and imaging, followed by illustration of their preclinical in vitro applications in body fluids and cells, ex vivo tissues and in vivo small animals and ending with a brief discussion of their clinical translation. After comparing the advantages and disadvantages of IR/Raman imaging with the other main modalities, such as magnetic resonance imaging (MRI), computed tomography (CT), positron emission tomography/single-photon emission-computed tomography (PET/SPECT), ultrasound (US) and photoacoustic imaging (PAI), the design of multimodal probes combining vibrational imaging with other modalities is discussed, illustrated by some preclinical proof-of-concept examples.

## 1. Introduction

Scientific and technological advances in the last decades have led to the development of a series of novel noninvasive biomedical imaging modalities, including X-ray computed tomography (CT), magnetic resonance imaging (MRI), positron emission tomography (PET), single-photon emission-computed tomography (SPECT), ultrasound (US), photoacoustic imaging (PAI), optical imaging (OI) and infrared (IR)/Raman imaging. The need of medical imaging to generate image contrast beyond that provided by morphological differences alone led to the development of the field of molecular imaging, whose objective is to image molecules and biomolecular processes of medical interest within living patients in a noninvasive manner and, in real time, enable the representation of an image as a function of the chemical identity [1]. This is in sharp contrast to conventional methods for obtaining chemical information from preserved tissue samples, such as histology. The molecules used may be natural molecules or synthetic probe molecules injected into a patient. Usually a contrast agent (e.g., a microbubble, a nanoparticle (NP), a metal ion complex or a radioactive isotope) is injected into a patient’s bloodstream, and an imaging modality (e.g., US, MRI, CT or PET) is used to follow its distribution in the body. This allows a quantitative in vivo characterization of biological processes at the molecular and cellular levels by localizing and measuring specific molecular targets or biochemical pathways that are associated with the anomalies at the basis of a given pathology. These molecular biomarkers are identified by their specific interactions with the targeted imaging molecular probe that accumulates at the disease site and produces a signal that can be detected and quantitated by the appropriate imaging modality [2]. These agents are traditionally small molecules containing an imaging reporter providing the signal to be detected, connected to a molecular targeting vector (organic molecule, peptide, protein, oligonucleotide, antibody, etc.). However, progress in nanotechnology have led to the development of a huge variety of precisely engineered nanoparticle-based targeted probes for in vivo imaging with optimized properties, including multimodality detection and theragnostic applications [3,4]. However, their slow accumulation in the disease site and excretion potentiate their toxicity, which can limit their competition with small-molecule agents [5].

Recently, molecular imaging techniques based on IR and Raman spectroscopies have rapidly emerged within the biomedical area for disease diagnostics, combining the high spatial resolution of optical microscopy with vibrational spectroscopies to provide chemical specificity based on the interaction of radiation with molecular vibrations within the tissues to produce specific spectral signatures [6,7,8,9,10,11,12,13,14,15,16]. In this article, we briefly present (a) the physical basis and technical features of vibrational (IR and Raman) spectroscopy and imaging, (b) some selected biomedical applications of IR/Raman imaging and (c) a comparison of the advantages and disadvantages of IR/Raman imaging relative to the other main imaging modalities, complemented by examples of applications of multimodality probes involving vibrational imaging techniques.

## 2. Introduction to Vibrational Spectroscopy and Imaging

Vibrational spectroscopy, which includes the infrared and Raman techniques, probes the intramolecular vibrations of molecular bonds during irradiation with light. It has many chemical, physical and biological applications, amongst which is the label-free, nondestructive analysis of cells and tissues searching for spectral signatures or spectral biomarker characteristic of the molecules present in the sample under study, which reflects their total biochemical composition. Both Fourier-transform infrared (FTIR) and Raman spectrometers can be coupled to optical microscopes. The recent improvements in instrumentation and the design of advanced multivariate data analysis techniques enabled the imaging of cells and tissues [6,7,8,9,10,11,12,13,14,15,16]. 

### 2.1. Physical Basis of IR and Raman Spectroscopy

**Figure 1:**
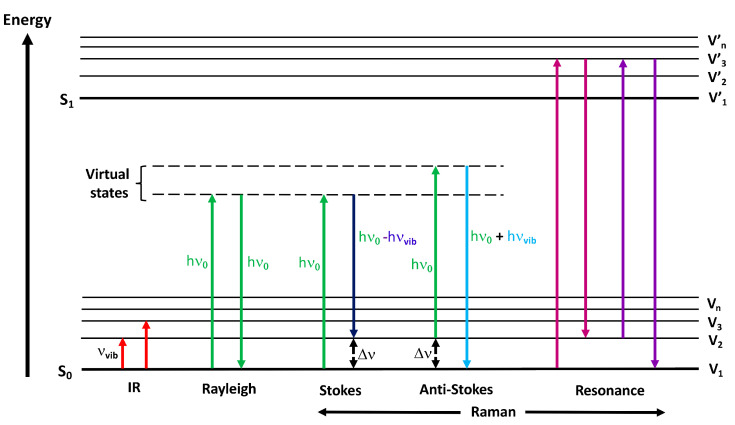
Jablonski energy diagram showing the transitions involved during infrared absorption, Rayleigh, Raman Stokes, anti-Stokes and Resonance Raman scattering. The vibrational states (V_n_) of a molecule in the ground electronic state (S_0_) can be probed either by directly measuring the absolute frequency (IR absorption) or the relative frequency or Raman shift (Stokes and anti-Stokes) of the allowed transitions. Resonance Raman also involves the vibrational states (V’_n_) of the excited electronic state (S_1_). Hν_0_ = incident laser energy, hν_vib_ = vibrational energy, ∆ν = Raman shift and ν_vib_ = vibrational frequencies.

When a sample is irradiated with light, it can be absorbed, transmitted, reflected or transflected (a combination of transmission and reflectance). Infrared spectroscopy can be defined as the study of the absorption properties of materials arising from changes of their molecular vibrational motions upon interaction with an IR source. During the light–molecule interaction, the electric field of the IR wave causes certain chemical bonds to enter a higher vibrational state due to the transfer of a quantum of energy when the energy of the incident radiation is the same as the energy difference between the two vibrational states involved (Figure 1). Only the chemical bonds with an electric dipole moment that changes due to the atomic displacements associated with the vibrations are IR-active. The corresponding transitions result in a spectrum with peaks/bands that can be interpreted qualitatively (peak position) and quantitatively (peak intensity/area, relative intensity), which is a molecular fingerprint characteristic of the composition of the material under analysis.

**Figure 2:**
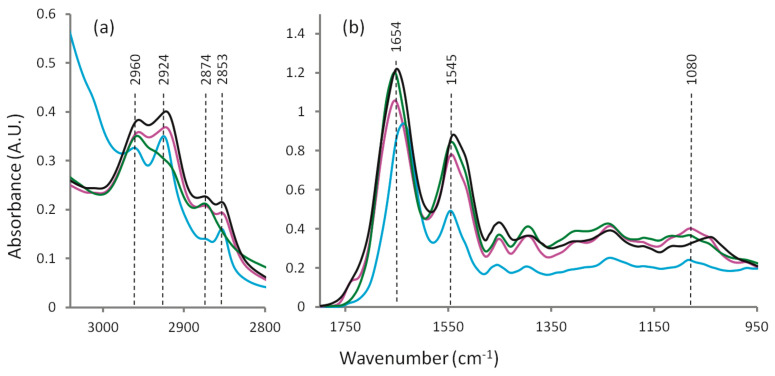
Average Fourier-transform infrared (FTIR) spectra from the liver tissue: hydrated tissue attenuated total reflectance (ATR) spectrum (blue), formalin fixed transmission spectrum (black), desiccator dried transmission spectrum (pink) and ethanol dehydrated transmission spectrum (green): (**a**) 3040–2800 cm^−1^ region and (**b**) 1800–950 cm^−1^ region. Reproduced from reference [17].

Biological samples are usually studied by FTIR spectroscopy in the transmission mode in the mid-IR region (approximately 4000–400 cm^−1^ or 2.5 to 25 μm), where the bands for most molecules occur, and the molecular absorbance is proportional to the concentration (Beer-Lambert’s law) in the absence of light scattering. Figure 2 shows a typical example of an FTIR spectrum of a liver tissue biopsy in various conservation conditions [17]. The spectrum can be divided into several regions where the main macromolecules—proteins, carbohydrates, nucleic acids and lipids—absorb. Table 1 lists the main spectral regions, the corresponding group vibrations and main contributing macromolecules. 

Despite its advantages, such as molecular specificity, FTIR spectroscopy suffers from some shortcomings that severely limit its application to biological samples—mainly, sensitivity issues and difficulties in the study of aqueous solutions. Sensitivity can be low—in particular, in thin samples such as monolayers—as a result of the Beer–Lambert’s law. This fundamental limitation can be overcome by strong signal amplification based on the plasmonic resonances present in nanoscale metallic particles [18], resulting in the phenomena of surface-enhanced infrared absorption (SEIRA) [19,20], using plasmonic chip-based technology to enable the in-situ monitoring of proteins and nanoparticle interactions in aqueous media at high sensitivity and in real time [21]. The other disadvantage of FTIR is that the absorption of water in the mid-IR region is very intense, as its OH-bending absorption is much stronger than any signal from the protein samples. This problem can be overcome partially by dehydrating the samples or, in the solution, by subtracting the water signal, limiting the path lengths to <10 μm and using relatively high protein concentrations (>20 mg·mL^−1^) to obtain appropriate signal-to-noise ratios (S/R). Alternatively, attenuated total reflectance (ATR) sampling is a promising technique for the analysis of aqueous biological samples. In this technique, an IR beam is focused at a set angle onto a crystal with a high refractive index, producing an evanescent standing wave resulting from internal reflections when it propagates through it. This wave reaches beyond the outer surface of the crystal and then into the sample by a few microns (0.5–5 µm), which is held in contact with it. The wave is either altered or attenuated in the parts of the spectrum where the sample absorbs energy and is guided back to the original IR beam, which leaves via the other side of the crystal and goes into the spectrometer’s detector, generating an IR spectrum. However, the appropriate use of this technique requires control of the contact between the ATR crystal and the sample, the beam penetration depth and distortion due to high refractive indices [15,22].

The spontaneous Raman effect, discovered nearly 90 years ago [23], consists of a two-photon inelastic scattering process following the interaction of a monochromatic radiation (e.g., laser source) with a sample. An incident photon, in the ultraviolet (UV), visible (VIS) or near-IR (NIR) spectral regions, induces, through its electric field, a change in polarizability, described as a deformation of the electron cloud of a molecule relative to its vibrational motion, which leads to an induced dipole moment, a partial positive/negative charge across a molecular bond existing in a virtual state (Figure 1). A photon is immediately emitted from the molecular bond, which returns to a ground state. During this interaction, both elastic and inelastic scattering processes take place. Most photons are emitted at the same frequency (energy) as the incident photon, corresponding to Rayleigh or elastic scattering. Raman scattering occurs when the photons are emitted at a frequency (energy) different from that of the incident photons. When the photons transfer energy to the molecules as vibrational energy, the energy (frequency) loss of the scattered photons corresponds to the vibrational energy levels of the molecules, which is known as Raman–Stokes scattering. When the incident photons receive energy from the vibrating molecules, their frequency (energy) increases, described as Raman anti-Stokes scattering. Figure 1 shows the transitions involved in these three processes. In spontaneous Raman, the Stokes scattering is generally used due to its higher sensitivity. 

**Figure 3:**
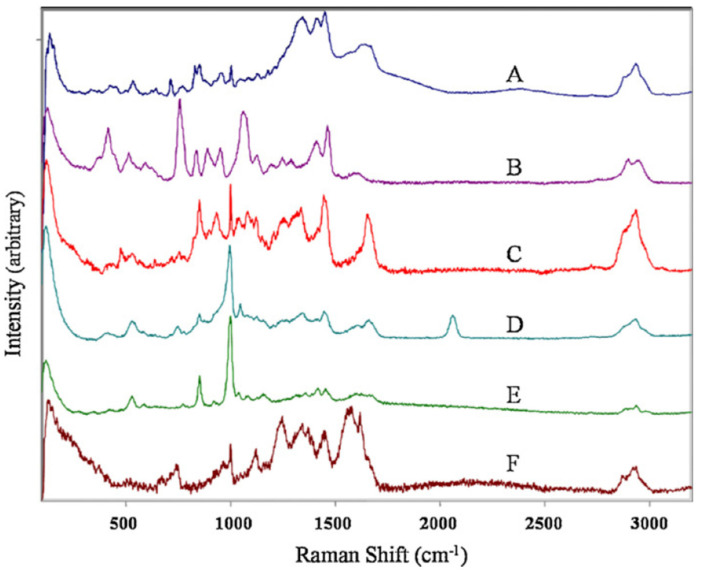
Raman spectra of human semen (**A**), canine semen (**B**), vaginal fluid (**C**), saliva (**D**), sweat (**E**) and blood (**F**). Reproduced from reference [24].

The very different mechanisms of the Raman and IR vibrational techniques endow them with some similarities and quite different characteristics, which are summarized here: (a) IR absorption results from changes in the dipole moment as the bonds vibrate, causing asymmetric and polar molecules to show strong IR spectra compared to more symmetric nonpolar molecules. Thus, most organic molecules show IR spectra with many overlapping bands, such as in FTIR spectra of cells or tissues. In contrast, Raman activity depends on changes in the polarizability tensor caused by molecular bond vibrations, and in general, only symmetric modes and nonpolar oscillating nuclei give strong Raman spectra. (b) Unlike FTIR, Raman spectroscopy does not suffer from water interference, as water is a very weak scatterer. Therefore, Raman measurements can be made directly from biofluids (Figure 3) and in vivo. (c) In both techniques, the bands occur at the same frequency for the corresponding active vibration modes, covering the range up to 4000 cm^−1^. (d) The Raman efficiency is quite low compared with FTIR, unless resonance or surface-enhanced Raman scattering processes are used. In fact, the spontaneous Raman signal is intrinsically quite weak because only one in ~10^7^ photons undergoes inelastic scattering, leading to Raman cross-sections (∼10^−29^–10^−30^ cm^2^) that are much lower than the usual absorption cross-sections of fluorophores (∼10^−15^–10^−16^ cm^2^). Therefore, the acquisition times of Raman spectra can be quite long. (e) Unlike in FTIR, the Raman spectra of biological samples collected with a visible laser can be dominated by a very broad fluorescence background, which needs to be removed mathematically to allow the observation of the sharp Raman peaks. [2,6,11].

The detection sensitivity of the spontaneous Raman signal can be increased, and the background fluorescence can be avoided using different Raman methods, which are based on several physical phenomena. The Raman signal enhancing mechanisms can be discussed by considering the factors that determine the intensity of the Stokes–Raman scattered light, I_Stokes_, given in Equation (1):

(1) $$I_{Stokes}\propto \;I_{0}\;{\left(\omega_{0}\;-\;\omega_{0,q}\right)}^{4}{|\alpha |}^{2}$$

where *I*_0_ is the incident light intensity, $\omega_{0}$ is the excitation frequency, $\omega_{0,q}\;$ is the vibrational frequency of the normal mode and α is the polarizability, which determines the response of the molecular electron cloud to the electric field E_0_ of the incident laser light. The Raman scattering intensity can be increased at shorter wavelengths due to its fourth power proportionality to the frequency of the incident laser light, but this is limited in biological samples due to the phototoxicity and degradation effects of UV light. Equation (1) also shows that the Stokes–Raman intensity can be enhanced by increasing either α or *I_0_*. These two quantities are used in the two most common enhancement techniques, Resonance Raman Scattering (RRS) and Surface-Enhanced Raman Scattering (SERS) [6,11,25]. In RRS (Figure 1), the incident photons of the laser used to excite the Raman spectrum are chosen to have an energy close to that of an intense electronic absorption band of a chromophore, which increases α and leads to an enhancement of the Raman scattering, so that some of the band intensities are increased by a factor of 10^3^–10^5^. RRS can selectively enhance certain chemical species by tuning the laser to certain wavelengths, such as aromatic amino acids at 227 nm or nucleic acids at 244 nm [26]. The SERS effect, first observed in 1973 [27], requires that an analyte is adsorbed or is in close proximity to a noble metal substrate, which is usually a roughened electrode, a functionalized metal nanosurface or a colloidal solution. The enhancement factor can be as high as 10^10^–10^11^, allowing the detection of single molecules [28]. The mechanism of SERS is explained by the electromagnetic theory, which proposes that the excitation of localized surface plasmons by the incident light increases the local electric field (E) provided by the surface, as well as the scattering intensity (I~|Ε|^2^), which is the maximum when the plasmon frequency is in resonance with the radiation. The scattering is provided only by the plasmon oscillations that are perpendicular to the surface. Alternatively, the chemical theory proposes the SERS effect through the formation of charge-transfer complexes from the metal surface to the adsorbing species, which also explains the RRS process. In fact, SERS can be combined with a chromophore in Surface-Enhanced Resonance Raman spectroscopy (SERRS) [29]. SERS has found many applications in chemistry and biology [30] and, recently, in biomedical research through the use of Ag and Au nanoparticle colloids as SERS substrates, which provide reproducible spectra from protein-free blood serum and plasma [31]. 

All the Raman scattering approaches discussed until now are known as spontaneous Raman processes, because the different vibrations of each molecule are excited independently, and their mode-to-mode phase relation is random. The resulting spectra are a superposition of the vibrations of all molecules that are excited by an interaction with the light source. Besides these, nonlinear Raman spectroscopic methods, based on the excitation of coherent molecular vibrations, have been developed. They can significantly enhance weak Raman signals and, thus, have been applied to the biomedical field. The two most common techniques are Stimulated Raman Scattering (SRS) and Coherent Anti-Stokes Raman Scattering (CARS) [12,16,32]. 

Controlling the sampling depth and location of the spectral information obtained in Raman spectroscopy allowed the subsurface analysis of biological tissues and other media with high turbidity, which opened the way to new biomedical applications. The most important methods in this area are Spatially Offset Raman Spectroscopy (SORS) and Transmission Raman Spectroscopy (TRS). SORS is based on the spatial separation of the Raman collection zone from the laser illumination zone on the surface of the sample. This approach suppresses the Raman and fluorescence contributions from the sample surface present in conventional Raman methods, allowing a deeper probing of the media that scatter light diffusely. TRS is a special case of SORS, where the laser beam and the Raman collection zone are completely separated by being on the opposite sides of sample. These techniques have been used, for instance, in the in vivo analysis of breast cancer. The sensitivity in through-tissue measurements can be increased using a combination of SORS with nano-tagged SERS particles (SESORS), allowing noninvasive measurements at depths of up to 45–50 mm in biological tissues [13].

In conclusion, IR and Raman spectroscopies are complementary, as they produce different fingerprints of the molecules present in a biological sample, depending on whether their bonds are Raman or IR-active, as certain vibrations that are allowed in Raman may be forbidden in IR and vice versa. A description of the fundamentals and applications of IR and Raman spectroscopy can be found elsewhere [33,34]. 

### 2.2. Basic Principles of IR and Raman Imaging

Vibrational (FTIR and Raman) micro-spectroscopy, or spectroscopic imaging, combines vibrational spectroscopy and light microscopy to visualize complex biomedical samples, such as cells and tissues, at the micron and the submicron levels. These techniques belong to the chemical imaging family, where the image contrast of the tissue or cell results from the chemical identity of the sample and not just from its morphology.

FTIR micro-spectroscopy combines the high spatial resolution of microscopy with IR spectroscopy to provide spatially resolved IR spectra. Since IR is not transmitted by glass, all the instrumentation optics, including the microscope objectives, are mirror-based. Nearly all measurements use an interferometer, where the observed time-dependent signal (interferogram) is converted to the spectral domain by a Fourier transform, carried out automatically using the instrument control software. IR sources can be (a) thermal, with low fluxes, which limit the obtainable signal-to-noise ratio (S/N), (b) synchrotron sources, which are 100–1000 times brighter, giving better S/N while maintaining high resolutions and (c) quantum-cascade lasers (QCLs), with a brightness up to three orders of magnitude higher than that a synchrotron, depending on the wavelength. IR detectors can be single-point, linear array (LA) or two-dimensional (2D) focal Plane Array (FPA) multi-channel detectors made of mercury cadmium telluride (MCT). Transmission, transflection or attenuated total reflection (ATR) detection modes can be used.

To obtain laterally resolved 2D IR spectra requires detection of the radiation on a specific region of a sample. This can be achieved in two different optical configurations: mapping techniques, in which the restriction of the radiation arriving at the sample plane is done with an aperture, and imaging techniques, where radiation segmentation at the detection plane uses FPA detectors. In the FTIR mapping approach, a grid on the sample area of interest is defined, and IR spectra are acquired sequentially from each grid point using a single-element detector and a motorized sample stage. The lateral resolution depends on the size of the aperture and the pixel size. Achieving a high lateral resolution near the diffraction limit of IR radiation requires small apertures (≤10 × 10 μm^2^), which severely restricts the IR flux that reaches the detector, leading to long acquisition times or low S/N when using a thermal source. When using small apertures, the sensitivity can be improved using IR synchrotron radiation. In the imaging approach, the entire field of view is illuminated (wide-field illumination) and imaged, without the need for scanning, on an array of IR-sensitive FPA detectors. Each individual detector in the array simultaneously collects data from a specific sample region within the field of view without using apertures. IR spectral images with an FPA can be generated by scanning the delay of a FTIR interferogram or by scanning the QCL laser wavelength. Wide-field scanning of a sample is possible in seconds, providing tens of thousands of spectra. The lateral resolution is determined by the magnification of the optical system and the size of the detector elements, reaching values close to the diffraction limit [14,15,35].

The diffraction limit for spatial resolution is often described using the Rayleigh criterion, where the spatial resolution *r* is given by Equation (2):

(2) $$r=\;\frac{1.22\;\lambda }{2\;\mathrm{NA}}$$

where *λ* is the wavelength of the exciting light, NA is the numerical aperture of the objective and NA = *n* sin *θ*, where *n* is the refractive index of the medium between the objective and the sample, and *θ* is the acceptance angle of the objective [14]. Under ideal conditions, the maximum achievable spatial resolution is approximately 1/*λ* (Abbe’s diffraction limit), which, for the fingerprint region, corresponds with a spatial resolution of between 2.5–5 μm. A transmission mode IR microscope using a FPA detector operates with a spatial resolution of 5 to 6 μm to ensure a sufficient S/N. This value is lower than the diffraction limit, which lowers the image quality. The diffraction limit at a given wavelength should decrease by increasing the NA, which can be obtained by increasing the *θ* or *n*. The latter methodology was developed by Kazarian using ATR imaging and a crystal having a high refractive index. The application of this technique to biomedical samples gave a four-fold improvement in spatial resolution [22].

The array of spectral data obtained by these methods can be used to obtain label-free chemical images or maps, showing the spatial distribution of specific biomolecules in a sample, either by plotting the change in the absorbance of a specific spectral band along the imaged area or by using a chemometric analysis to group the spectra into clusters that belong to the different chemical compositions in the sample [36]. Data can be obtained from a variety of samples, such as fixed or dried cells, tissue biopsies or live cells. The different FTIR imaging approaches used depend upon the type of biomedical application, the size of the sample and the matrix that surrounds it and the concentration of the molecule to be analyzed.

Raman imaging combines Raman spectroscopy coupled to a microscope with a motorized sample stage, a Notch filter for Rayleigh scattering suppression, a solid-state laser source and a monochromator for the analysis of the scattered light and charge-coupled device (CCD) camera. The incident light is focused on the biological sample through an objective lens with a high numerical aperture (NA) to provide the resolution corresponding to the diffraction limit. The diffraction limits for the lateral and axial spatial resolution, *δ*_lat_ and *δ*_ax_, are given by Equation (3):

(3) $$\delta_{\mathrm{lat}}=\;\frac{0.61\;\lambda }{\mathrm{NA}}\;\;\;\;\;\;\;\;\delta_{\mathrm{ax}}=\;\frac{2\;\lambda n}{{\left(\mathrm{NA}\right)}^{2}\;}\;\;\;\;\;\;\;\;\;\;\;$$

where *λ* and NA were defined above, and *n* is the refractive index. 

Three different spontaneous Raman imaging methodologies were developed: (i) point mapping and (ii) line mapping as the serial imaging approaches and (iii) direct or wide-field Raman imaging as a parallel imaging approach. In point mapping, a Raman spectrum is acquired at each spatial location in a confocal configuration to generate a hyperspectral data cube, whereas in-line mapping a Raman spectrum is obtained for each row of the CCD detector, corresponding to a spatial location along the length of the laser line. In wide-field imaging, the whole sample field of view is illuminated with a laser light and analyzed in parallel. Discrete light frequencies are analyzed as a function of time by employing a wide-aperture optical filter. For the in vivo data acquisition, Raman systems are coupled to fiber-optical probes, e.g., in fiber array spectral translation (FAST) [7,8,12,16,35].

Comparing spontaneous Raman with FTIR imaging, the former can use visible laser light, which allows to obtain a lateral resolution <0.5 μm. This spatial resolution is similar to that of confocal fluorescence microscopy and is better than that of FTIR, which is around 2–10 μm, depending on the wavelength. As water has negligible Raman scattering, the technique is much more suitable for the analysis of living cells than FTIR, as long as the laser light power is minimized to avoid cell damage. However, FTIR imaging using a FPA can study larger areas of cells or tissues in a more reasonable time than Raman imaging, where usually only small areas can be analyzed (20 μm × 20 μm). Due to its low sensitivity, only a poor S/N ratio is usually obtained, especially for nonchromophoric asymmetric molecules. This leads to longer acquisition times, especially for thin (<1 μm) biological samples. Finally, although both FTIR and Raman are considered to be nondestructive, Raman imaging uses intense VIS, UV and NIR laser excitation sources, which can lead to localized thermal heating and photodecomposition. 

The low sensitivity of spontaneous Raman techniques has led to recent advances in nonlinear Raman microscopic methodologies, such as CARS and SRS microscopy, as well as SORS imaging, which discriminates against surface layer-generated fluorescence emission. Other techniques are based on the surface plasmon enhancement of the electric field intensity of the excitation beam, either using Au/Ag NPs in SERS imaging or using an Au/Ag-coated metallic tip, e.g., a metallized atomic force microscopy (AFM) tip, in tip-enhanced Raman spectroscopy (TERS) imaging. TERS achieves a 10^7^-fold amplification in the Raman signal, with sensitivity down to the single-molecule level. Its spatial resolution, approximately of the size of the tip apex (typically 20–30 nm), is by far better than Abbe’s diffraction limit of *λ*/2 [7,8,12,16,35].

## 3. Overview of Biomedical Applications of Vibrational Imaging

In this section, a brief overview is presented of biomedical applications of IR/Raman spectroscopy and imaging, from in vitro body fluids and cells through ex vivo tissues and organs to in vivo preclinical animal studies. The large body of reported studies can be found in exhaustive review articles [10,16,37]. 

### 3.1. In Vitro Studies

In vitro studies of body fluids, such as blood, plasma, bile, cerebrospinal fluid (CSF) and amniotic fluid, have been performed using IR and Raman spectroscopy and the multivariate data analysis. With such metabolic fingerprinting (metabolomics) studies, molecular biomarkers of cancer and other pathologies, such as diabetes and arthritis, could be defined [6,9,11]. The presence of water in the samples is not a problem for the Raman analysis but is strongly limiting in the normal mid-IR region due to strong water bands at 3400 and 1640 cm^−1^. This can be avoided by using near infrared (NIR) spectroscopy, which is based on molecular overtone and combination vibrations [6]. As these transitions are forbidden, the molar absorptivity in the near-IR region is quite small. One advantage is that NIR can penetrate much deeper into a biological sample than mid-IR light.

### 3.2. Ex Vivo Cells and Tissues Studies

IR and Raman spectroscopy have also been used extensively in living cell studies for metabolic fingerprinting using the multivariate data analysis in the diagnosis of diseases such as leukemia, cervical, prostate and other types of cancers [6] and for studying drug–cell interactions in pharmaceutical applications [38,39]. These spectroscopies were also used in biomedical studies of different kinds of ex vivo tissues and biopsies [35] from many types of cancers [6], including breast cancer [8], to neurological conditions such as multiple sclerosis [40]. Likewise, IR and Raman imaging have found many biomedical applications through the analysis of ex vivo tissues and biopsies at the subcellular level, such as in spectral histopathology (SHP), which can be compared with gold standard histology (using hematoxylin and eosin (H&E)-stained tissues) [8,14,15,16,36] and drug diffusion into ex vivo tissues [15,16].

The main challenges of FTIR imaging of living cells and ex vivo tissues are the background scattering, the limited spatial resolution, the relatively low limit of detection and the strong background absorbance from water (70%). Eukaryotic cell sizes and tissue features are typically between 10–100 μm and cell organelles between 1–10 μm. As the wavelengths used in IR spectroscopy (2.5–25-μm range in the mid-IR region) are very similar to the size of biological cells, they can cause intense Mie scattering (in contrast to Rayleigh scattering for particles smaller than the wavelength of scattering light), which results in an undulating baseline of IR spectra of biological samples. Additionally, a very distorted line shape is obtained due a sharp decrease in absorption at 1700 cm^−1^ (the “dispersion artefact”). These combined effects lead to variations in the relative peak intensity, which is particularly important for the amide I and amide II bands, as they are commonly used to quantitate proteins and identify their secondary structures. Mie scattering also results in shifts in the observed frequency of spectral bands. All these spectral artefacts can be reduced or corrected by algorithms. Most live cell studies use transmission FTIR imaging with a synchrotron source, which provides diffraction-limited spatial resolution images with high S/N spectra. Therefore, the spatial analysis of living cells is limited to the measurement of differences between the cytoplasm and nucleus regions and investigation of drug uptake into cells [14]. 

As discussed in the previous section, spontaneous Raman imaging of cells and tissues has negligible water interference, somewhat better spatial resolution and a smaller field of view than FTIR imaging. However, its main limitation is a low sensitivity, which can be overcome by nonlinear Raman techniques (CARS and SRS microscopy), surface plasmon-based techniques, such as SERS and TERS imaging, and Resonance Raman (RR) imaging. The first techniques have been applied to a variety of pathological tissues, such as brain cancer and breast cancer. For tissues containing high concentrations of natural fluorophores, such as carotenoids and hemoglobin, normal, RR imaging has been used, while UV light-excited RR (UVRR) has been used to detect RNA/DNA [9,12,16].

### 3.3. In Vivo Small Animal Studies

Strategies for in vivo small animal Raman imaging have also been described. One approach used SERS Raman-active nanostars and nanospheres and single-wall carbon nanotubes (SWNT) to perform deep-tissue Raman imaging in small animals [12,41]. Pharmacokinetics, multiplexing and in vivo tumor targeting of NPs were demonstrated [12]. For example, SERRS nanostars were used in the colon cancer imaging of mice, with the delineation of tumor margins both at the primary tumor sites and metastasis during image-guided surgery [42]. Alternatively, fiberoptics Raman probes have been used in small animal imaging [9]. The potential of this technique for the in vivo diagnosis of brain tumors was demonstrated by obtaining Raman spectral maps over small areas (3.6 mm × 3.2 mm), which allowed the label-free diagnosis of induced metastatic brain tumors in mice with an accuracy of ~250 μm [43].

## 4. Comparison of IR and Raman with Other Molecular Imaging Techniques and the Use of Multimodality Probes

### 4.1. Comparison of the Main Molecular Imaging Techniques

The physical basis and the contrast mechanisms operating in IR/Raman imaging techniques were discussed in Section 2. In order to compare them with the other main imaging modalities currently used in preclinical research and in clinical practice, a brief overview of those modalities is presented here. However, due to the complexity of the underlying physical principles of each imaging modality, an in-depth approach to each of them is beyond the scope of this review. Table 2 summarizes the main characteristics of the imaging modalities, showing their advantages and limitations [2,3,44,45]. 

MRI is based on the principles of nuclear magnetic resonance (NMR), whereby tomographic images are obtained after protons absorb the energy of radio frequency (RF) pulses when placed in an external magnetic field; the resultant evolving macroscopic spin polarization induces a RF signal in a tuned radio frequency coil and thereby is detected while magnetic field gradients localize the polarization in space. Image contrast can be obtained mainly on the basis of the differences in spin–lattice (*T*_1_) and spin–spin (*T*_2_) relaxation times of the water and lipid protons present in various tissues and organs, although the differences in water proton densities, diffusion constants and flow characteristics can also be explored [46]. The outstanding diagnostic capabilities of MRI result mainly from the high spatial resolution of the images produced noninvasively and without the use of ionizing radiation. MRI provides images with great soft tissue contrast, allowing the detailed visualization of organs and tissues, with rich anatomical and physiological information. However, in vivo molecular information is more difficult to obtain by MRI due to its poor sensitivity. Other important constraints of this technique are high costs and long examination times. The capacity of MRI to distinguish small areas of diseased tissues from healthy ones can be enhanced with the use of contrast agents. These molecules, metal complexes or nanoparticles affect the intensity of the MRI signal by shortening either the *T*_1_ or *T*_2_ relaxation times of the water protons around them. They are divided into paramagnetic (*T*_1_ or positive) agents, such as highly stable Gd^3+^ or Mn^2+^ complexes with polyaminocarboxylate chelators, and superparamagnetic (*T*_2_ or negative) agents, such as small superparamagnetic iron oxide NPs (SPIONs) or ultrasmall superparamagnetic iron oxide NPs (USPIONs) [47,48,49]. 

X-ray computed tomography (CT) was introduced to clinical practice in 1972 and became one of the most frequently used imaging techniques. A CT image is obtained using an X-ray beam source and the corresponding signal acquisitions taken from different angles, which are processed to reconstruct 3D images. When an X-ray beam travels across the body, its interaction with the tissues causes some of it to be absorbed or scattered, and the transmitted beam intensity is attenuated. This selective attenuation by the tissues increases with the linear attenuation coefficient (*μ*), the density (*ρ*) and the thickness of the materials present in the tissues they cross and, also, depends on the X-ray energy (*E*) [50]. CT images provide great anatomical information, being more sensitive to denser tissues or bones than to soft tissues. Bones appear white on an X-ray image because absorption is greater in bone, containing materials with atoms of a high atomic number (*Z*), than in soft tissue (effective *Z*~7.5). The contrast in soft tissues can be improved using contrast agents made of high Z materials, such as barium (*Z* = 56) sulfate suspensions, iodinated compounds (iodine has *Z* = 53) or gold (Au, *Z* = 79) NPs. The main advantages of CT are their relatively short scan times and low costs, and thus, high availability in hospitals and clinics, and good resolution. However, its main limitation is the overexposure to potentially harmful ionizing radiation and low sensitivity [51,52,53]. 

PET and SPECT are highly sensitive diagnostic imaging techniques used in clinical nuclear medicine, providing the visualization of biological processes at the molecular and cellular levels, which allow to image pathophysiological processes and to monitor the therapeutic effects in vivo. Both modalities require the injection of molecules labeled with radioisotopes that decay by the emission of *β*^+^ particles (positrons) or *γ*-rays, respectively. These radiopharmaceuticals are administrated in tracer quantities and are then distributed, metabolized and excreted from the body, depending on their physiological function. During the decay process, the *β*^+^ or *γ* particles are detected to produce an image. The radionuclide imaging agents should have half-lives (*t*_1/2_) that match the half-life of the tracer molecule used in each application. In PET, the positron-emitting radionuclides (e.g., ^11^C, ^18^F, ^68^Ga and ^89^Zr), with relatively short half-lives, are visualized and quantified in living subjects through the detection of the annihilation products of the emitted positron with an electron from the surrounding tissue, with the simultaneous production of two 511-keV *γ*-rays that are emitted in opposite directions to one another. Detectors on opposite sides of the patient, being part of a circular array surrounding him, detect simultaneously a large number of these pairs of *γ*-rays (coincident events), which can be used to reconstruct a PET image that contains quantitative information about the distribution of radioactivity within the tissue. The radioisotopes used in SPECT imaging (e.g., ^99m^Tc and ^111^In) decay directly into *γ*-rays, with generally lower energies and longer half-lives than those used in PET. After the emission from the radionuclide, the single photons travel through the tissues and are detected by a circular array of gamma cameras to obtain the images. Although the high sensitivity of these techniques is a very important asset in their clinical applications, the anatomical information provided by the images obtained is limited by their low spatial resolution. Besides this, the other main drawbacks of the nuclear medicine imaging techniques are the exposure to ionizing radiation, the high costs and, often, the use of short imaging times due to the short half-lives of the radioisotopes [53,54,55].

Optical imaging (OI) is a noninvasive imaging technique that uses nonionizing radiation, such as visible, ultraviolet and infrared light, to obtain the molecular and physiological information of cells, tissues, organs or whole bodies, playing an important role in the preclinical research and medical diagnosis [56]. It includes several techniques, such as fluorescence and bioluminescence imaging, optical coherence tomography (OCT) and endoscopy. In fluorescence imaging, the light emitted by excited exogenous probes is detected in a spatially resolved manner by a camera. OI is simple to operate, safe, cost-effective and sensitive, providing real-time imaging, subcellular resolution using nM-probe concentrations and the use of multiple probes with different properties, which allows multichannel detection. The main disadvantages are the poor tissue penetration depth, due to strong light absorption, background signal due to light scattering and autofluorescence, affecting the image-optical contrast and low photostability of some organic dye probes. These features hinder the clinical translation of some of these techniques [57,58,59]. However, fluorescent probes are used for image-guided tumor surgery, while near-infrared (NIR) fluorescent organic fluorophores and luminescent NPs, such as NIR quantum dots and lanthanide-doped upconversion NPs (UCNPs), are being developed to improve tissue penetration [57,58,59,60,61,62,63]. With such a variety of optical probes available, many studies of cellular processes using fluorescence confocal microscopy and imaging have been carried out. Fluorescence imaging was also used for in vivo small animal whole-body tissue studies, mostly based on the planar detection of fluorescent light, using epi-illumination or the transillumination techniques. Fluorescence molecular tomography (FMT), which maps the 3D distribution of the probe noninvasively, overcomes the limitations of the above techniques to provide quantitative information on probe distribution. This results from the nonlinear dependence of the measured signal intensity on the depth of the probe location [64]. 

In ultrasound (US) imaging, pulses of ultrasound waves are produced and emitted by an ultrasonic transducer, which are transmitted through the body and interact with organ boundaries and complex tissue structures, producing echoes due to reflection or scattering, which are received by the same transducer. Theses echoes are processed digitally to reconstruct an image, representing in a gray scale the two-dimensional cross-section of the body with a contrast that reflects the distance, size and density of each tissue structure originating the echoes. Three-dimensional US images can also be obtained by moving the transducer over the surface of the body under study. US can be acquired in the Doppler mode, in which the changes in the pitch and phase of the sound waves are used to obtain the velocity and direction of movements inside the body, such as blood flow in the heart and vasculature. The contrast in US images is due to the differences in the acoustic impedance (echogenicity) of the imaged tissues. A spatial resolution of ∼0.2–1 mm is obtained for the typical sound wave frequencies (above 1 MHz) applied in clinical use for ultrasound waves generation and detection [65,66]. US is one of the most widely used modalities for imaging soft tissues, providing the diagnosis and staging of many pathologies. It is safe (noninvasive and nonionizing), accessible and relatively cheap, with high spatial resolution and excellent temporal resolution, which can be used for real-time diagnostics, as well as for therapeutic purposes (High-Intensity Focused Ultrasound—HIFU). However, its main limitations are the limited tissue penetration, whose depth decreases when the spatial resolution increases, high background noise and, sometimes, insufficient acoustic contrast between the tissue constituents. These limitations lead to the development of exogenous acoustic ultrasound contrast agents (USCAs) with high echogenicity [67,68,69]. One example is gas-filled microbubbles (∼1–4-μm diameters), which, upon intravenous injection, are retained in the vascular space [68]. Several other types of nanometric USCAs have been developed [66,68]. 

Photoacoustic imaging (PAI) is based on the photoacoustic effect first described in 1881 by Alexander G. Bell, which consists of the generation of sound waves after light absorption within a media. When an ultra-short pulsed laser irradiates a tissue, light absorption by an endogenous or exogenous tissue chromophore generates heat that is released to the immediate environment, causing its rapid thermoelastic expansion and contraction and leading to a high-amplitude, broadband acoustic wave. These ultrasound waves can be detected using standard US transducers, and photoacoustic images can be reconstructed from them [70]. Therefore, PAI has high optical contrast at the higher spatial resolution and depths (up to 7 cm) that are typical of US. It also has many molecular imaging applications, based on the molecular structure dependence of the optical absorption of the chromophores used. The PAI contrast depends on the difference of the photoacoustic signal intensity of the tissues, which is proportional to the optical-to-acoustic conversion efficiency of the media. This is determined by the number of photons absorbed by the chromophore and the ability of the tissue to convert it to heat that generates the acoustic wave [70]. PAI can use endogenous contrast agents present in tissues, such as hemoglobin, melanin and lipids, as well as exogenous dyes or metal-based NPs with good optical or magnetic properties, such as gold NPs, semiconducting quantum dots, carbon nanomaterials or magnetic iron oxide NPs [66,70]. Small blood vessels can be imaged using hemoglobin as a PAI probe [71]. The dependence of the PAI signal on the wavelength of the laser used can be exploited to obtain functional information by photoacoustic microscopy, such as blood oxygenation levels and the presence of atherosclerotic plaques in vessels [72], or in applications of multispectral optoacoustic tomography (MSOT) [73].

Raman imaging is label-free, nondestructive and with a very high and rich chemical specificity. This is in contrast to other optical techniques, such as phase-contrast microscopy and confocal fluorescence, which either rely on labeling or tissue physical properties, such as refractive indices or autofluorescence, and they also have broader, less-resolved bands. It uses low energy light, which usually does not cause sample photobleaching or ionization, making it particularly suitable for the analysis of biological samples and live cells. However, due to its low sensitivity, in vivo Raman imaging is challenging unless certain metallic NPs (e.g., Ag, Au and Cu) are used to amplify the signal in deep tissues. The SERS (and variants, such as SESORS) technique amplifies the Raman signal by a factor of ~10^10^, enabling detection of the nanoparticles up to a 1–5-cm tissue depth at fM concentrations. The high sensitivity of SERS, the lack of toxicity of its imaging NPs and its multiplexing capabilities make it a promising modality for future clinical applications. However, the still-limited penetration depth and difficulties with imaging large fields of view and in implementing tomography limit whole-body deep tissue imaging with SERS-based techniques. Thus, the most promising future clinical applications of SERS imaging will be those avoiding deep tissue penetration, such as endoscopy [2,66].

The basic characteristics of the main imaging modalities summarized in Table 2, and the consequent technical limitations, clearly highlight their advantages and disadvantages [2,66]. Spatial resolution increases from PET/SPECT, through MRI, CT, US and OI, to PAI, OTC, IR and Raman. On the other hand, the sensitivity is extremely high in SERS–Raman and PET/SPECT, high in OI and PAI, medium in US and MRI and low in CT. MRI has the lowest temporal resolution (scan time), but this is compensated by an unlimited penetration depth, like CT and PET/SPECT. This property is below 1 mm for IR and Raman (except with SORS) and the mm–cm range for US, PAI and OI. The image contrast of soft tissues is optimal in IR and Raman and is better for MRI, PET, SPECT and PAI than for CT and US. Additionally important are the possibility of multiplexing in OI, PAI and SERS-Raman; quantitative imaging in SPECT; whole-body imaging in PET/SPECT, MRI and CT and real-time imaging of US, IR and Raman. Other important characteristics dictate the current clinical use of the different modalities. US, MRI, OI and PAI have good safety profiles, which are poorer for CT and PET/SPECT, due to the use of ionizing radiation. US, OI and PAI are easier to use than CT, MRI and PET/SPECT. The throughput capacity is high for PAI; medium for US and OI and low for CT, MRI and PET/SPECT, while the cost is very high for PET and MRI; high for CT and SPECT and low for US, OI and PAI. Consequently, US, CT, MRI and PET/SPECT are currently used clinically, while OI, PAI, IR and SERS–Raman have emerging clinical utility.

The intrinsic characteristics of the main imaging modalities (Table 2) also have strong implications in limiting preclinical molecular imaging and clinical practice [2,3,66]. For instance, the high spatial resolution of MRI is often not enough to allow the in vivo imaging of biomarkers, due to the low intrinsic MRI sensitivity. In fact, The MRI CAs only produce a detectable image contrast enhancement at a relatively high local concentration (about 10 μM), which can saturate the targeted cell receptors. This is because such local concentrations are considerably higher than the typical cellular concentration of a particular receptor, which is in the nM–μM range per cell volume (corresponding to 10^5^ receptors per cell). The problems related to the low intrinsic sensitivity of MRI can be addressed by using targeted NPs capable of delivering a high payload of magnetic centers concentrated in a small volume. The high sensitivities of radionuclear (PET/SPECT) and optical imaging modalities (10^−10^–10^−12^ M) and of SERS–Raman (10^−12^–10^−15^ M) are much better-suited to address this problem.

These and other examples illustrate the frequent difficulty to obtain accurate and reliable information of pathological sites using a single imaging modality. This issue was addressed through the design of NPs containing reporters capable of multimodality detection. In these NPs, imaging modalities with high sensitivity (such as PET and OI) are usually combined with others providing high spatial resolution (MRI, CT, etc.), such as in NPs for PET/CT or PET/MRI. The limited tissue penetration of OI, PAI or SERS–Raman probes can be compensated by their conjugation with MRI/US/PET probes in a single NP. However, the different sensitivities of the modalities combined in one NP should be compensated by using the appropriate relative concentrations of their active probe centers in the combined nanoplatforms. 

Recently, a large variety of NPs has been developed as probes for multimodal imaging [2,3,44,66,74,75]. Combined hybrid images of CT/MRI with PET or SPECT can complement anatomical and molecular/functional information, enabling the noninvasive and quantitative assessment of drug-targeting mechanisms and efficiency [76]. Another important aspect is the recent development of virtually all hybrid preclinical and clinical imaging systems, such as SPECT/CT, PET/CT and PET/MRI scanners, allowing simultaneous (rather than sequential) whole-body imaging. This multimodality approach provided integrated theragnostic platforms [4] that could be used in preoperative diagnosis and intraoperative NIR-I/II fluorescence-guided surgery, as well as postoperative monitoring [77].

### 4.2. Applications of Multimodality Probes Involving Vibrational Imaging Techniques

**Figure 4:**
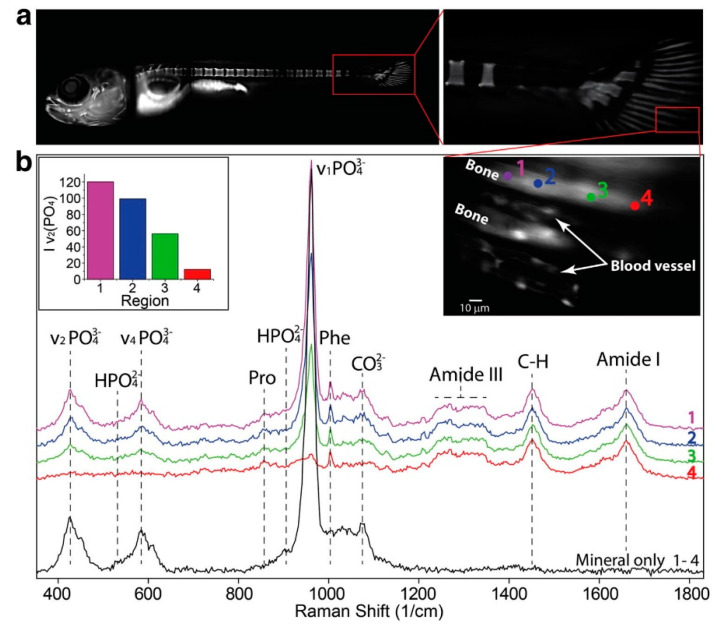
Correlative fluorescence–Raman imaging of zebrafish fin bone maturation. (**a**) Low-resolution (10×) fluorescence image of zebrafish stained with calcein green, with high-resolution (60×) details (right inset in panel b) of a representative fin ray region where Raman spectra (**b**) of progressively mineralized bone tissues were acquired (numbered 1–4). (Left inset in panel b) Integral of the orientation independent mineral band (v_2_) where a clear drop of the mineral content can be observed. Reproduced from reference [83].

The diagnostic performance of Raman imaging was improved using different approaches [16]. One of them was to develop dual-mode instruments and techniques to combine the biochemical specificity of Raman spectroscopy and imaging with the sensitivity and rapid screening capability of other label-free high-resolution optical imaging techniques, such as phase contrast microscopy [78], autofluorescence [79,80] and optical coherence tomography (OCT) [81,82] of cells and pathologic tissues. Confocal Raman spectromicroscopy and fluorescence imaging were also combined to study the early stages of bone formation in zebrafish (*Danio rerio*) larvae, used as a model organism to study vertebrate development (Figure 4) [83]. The Raman spectra in Figure 4b show characteristic bands of the organic protein extracellular matrix (e.g., amide I, amide III, Phe and C-H) and the inorganic mineral contents (e.g., v_1_, v_2_ and v_4_ of PO_4_^3−^ and CO_3_^2−^), whose intensity change reflected the chemical changes occurring along the bone maturation process. Histopathology, Raman microscopy and electron microscopy were also combined to image microcalcifications (MCs) in frozen human breast cancer tissue. The obtained μ-scale resolution correlative maps of the crystalline phase, trace metals, particle morphology and organic matrix chemical signatures revealed the heterogeneity of the mineral–matrix pairings that are present in the MCs [84].

In another study, polarized Raman spectroscopy (PRS), scanning acoustic microscopy (SAM) and synchrotron X-ray phase contrast nanotomography (SR-nano-CT) were combined to investigate the chemical composition, the complex architecture and the resulting elastic properties of a healthy femoral bone at the level of single-bone lamellae and entire structural units [85]. Raman spectroscopic and matrix-assisted laser desorption/ionization (MALDI) mass-spectrometric imaging were also combined to address the molecular heterogeneity of ex vivo cancer tissues. A Raman spectroscopic-based spectral histopathology (SHP) of a larynx carcinoma tissue was confirmed with MALDI imaging, detecting different tissue types and metabolic states, which allowed a better characterization of epithelial differentiation and dysplasia [86]. 

**Figure 5:**
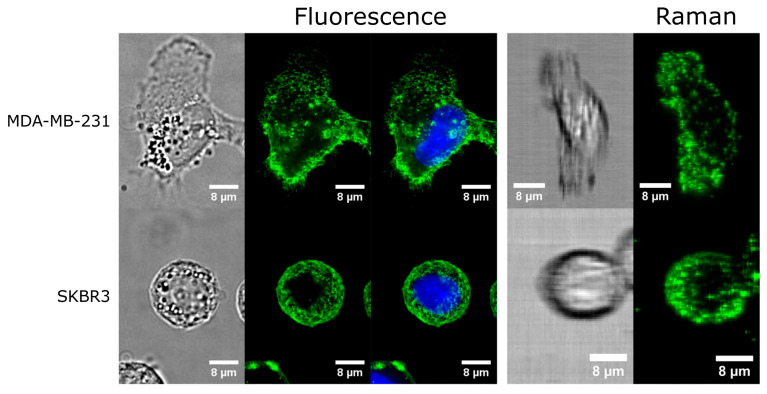
Comparison of Surface-Enhanced Raman Scattering (SERS) and fluorescence images. **Top**: Fluorescence and Raman images of MDA-MB-231 cells stained with CD44 fluorescence and SERS conjugates, respectively. **Bottom**: Fluorescence and Raman images of SKBR3 cells stained with HER2 fluorescence and SERS conjugates, respectively. All images are accompanied by brightfield images for reference. Blue indicates the nuclei of the cells. Scale bars are 8 μm. Reproduced from reference [88].

A different approach to cell and tissue multimodal imaging is a label-dependent one, which exploits the multimodality and multiplexing potential of SERS-active NPs capable of two or more readout modalities. The most common combination is that of fluorescence and SERS to obtain dual-modality nanoprobes. Coating AuNPs with a Raman reporter and a silica spacer shell having an embedded fluorescent dye can be used to prevent fluorescence quenching. Its coating with a further silica shell allowed conjugation of the NPs with CD24 and CD44 antibodies. The fluorescence–SERS duplex imaging of MDA-MB-231 breast cancer cells, which co-express CD24 and CD44 markers at their surfaces, confirmed their colocalization in the cells. This was one of the first demonstrations of targeting and imaging the local distribution of specific cancer markers on living cells, illustrating the potential of antibody-modified dual tags [87]. Despite the attractive properties of SERS NPs as a cell-labeling alternative to fluorescent probes, their sizes, shapes, surface properties and formation of a protein corona affect their interactions with cells. When using SERS NPs antibody conjugates, antibody multivalency should also be taken into account to validate them for live cell imaging applications. A study comparing the behavior of SERS NPs conjugated with HER2 and CD44 antibodies with the corresponding fluorescent-labeled antibodies, in the targeting of their receptors in breast cancer cells, Raman images demonstrated cell labeling with a high specificity that correlated well with fluorescent labels (Figure 5). However, monitoring surface biomarker expressions and the dynamics of the labeled live cells showed that the NPs were taken up by the cells and were compartmentalized into different cellular regions, with very different cell distributions from the fluorescent antibody conjugates. Thus, the cell internalization and trafficking of NPs can limit the use SERS NP-antibody conjugates to study cell dynamics [88].

Another bimodality study avoided antibody conjugates using NPs made of an Au core coated with the Raman reporter wrapped with an organosilica shell enriched with a fluorescent label. These SERS NPs were taken up into the cytoplasm of HeLa cells via a non-receptor-mediated endocytosis pathway, and their intracellular localization was imaged by fluorescence and SERS imaging [89]. The limitations of organic dyes as fluorescent probes, such as low photostability and broad emission bands, can be overcome by their substitution by quantum dots (QDs) or lanthanide-based upconversion NPs (UCNPS), which have narrow emission bands. The first study with upconversion fluorescence (UCF)-SERS dual-mode NPs for live cell and in vivo animal imaging used NaYF_4_:Yb,Er UCNPs@SiO_2_ as the fluorescent core, Ag NPs grown on their surface as the Raman reporter and a denatured bovine serum albumin (BSA) coating to provide stability and biocompatibility. The dual SERS/fluorescence imaging capabilities of the NPs were successfully evaluated in vitro with MCF-7 cells in vivo with mice using NIR laser excitation [90].

All the examples described above used fluorescence and SERS to localize the tags using the same information. A further development was to combine SERS tags with complementary functionalities and modalities to obtain information that is not achieved by SERS. An example of this was the use of magnetic SERS NPs for combined SERS/fluorescence imaging. Fe_3_O_4_ cores were first encapsulated into an inner layer of silica and then coated with a second layer of Au nanoshell (Fe_3_O_4_@SiO_2_@Au). Adjustment of the Au shell thickness can tune the surface plasmon resonance (SPR) of the Fe_3_O_4_@SiO_2_@Au NPs from the visible to the NIR region. After this, the NPs were covered with a third layer of Raman reporters to provide a SERS signal and coated again with an outer layer of fluorescent dye-doped silica. This onion-like multimodal nanoplatform could be taken up by Hela cells, with an enhanced uptake in the presence of a magnetic field. Fluorescence and SERS signals could be observed separately by exciting the NPs at different wavelengths, such as 515 nm and 633 nm, as proven by both fluorescence and SERS imaging [91]. A trimodality imaging nanoprobe (SERS, MRI and PAI) containing an Au core surrounded by a thin Raman active layer protected by a silica coating, which was functionalized with Gd(DOTA) (DOTA = 1,4,7,10-tetraazacyclododecane-1,4,7,10-tetraacetic acid), demonstrated its potential for more accurate brain tumor imaging and resection in living mice. MRI allowed preoperative detection and surgical planning. PAI, with its relatively high resolution and deep tissue penetration, could guide intraoperative tumor resection. The ultra-high sensitivity and spatial resolution of Raman imaging was then used to remove residual microscopic tumors. Finally, the examination with a Raman probe ex vivo and a histological correlation validated that Raman imaging was accurately delineating the brain tumor margins [92]. A limiting example, exploiting the multimodality and multiplexing potential of SERS NPs, was the development of theragnostic NPs active in five modalities: namely, SERS, MRI, CT, two-photon luminescence (TPL) and photothermal therapy (PTT). Au nanostars were covered with a Raman reporter for SERS, PTT and CT detection, then coated with PEG and a silica shell, which was conjugated with Gd(DOTA) as the MRI contrast agent. The potential of the nanoplatform for preoperative tumor scanning (MRI and CT), intraoperative tumor detection (SERS and TPL) and PTT as a postoperative treatment was demonstrated using BT549 cancer cells [93]. Besides the previous example, the theragnostic potential of SERS NPs was further explored, e.g., for SERS-monitored intracellular redox-triggered drug delivery [94] and cancer PTT [95].

### 4.3. Clinical Applications 

The applications of vibrational spectroscopy and imaging that can be translatable to clinical medicine have been recently reviewed and are summarized and classified in three areas [96,97]. The first one is Raman spectroscopy for intraoperative and in vivo diagnostics, such as the use of in vivo Raman fiber probes; deep Raman spectroscopy using TRS and SORS techniques to study tissues like skin and bone; endoscopic diagnostics of pathologies of hollow organs in the gastrointestinal (GI) tract (larynx, esophagus, stomach and colon) or in lungs (bronchoscopy) based on fiber optics Raman probes [16]; intraoperative Raman spectroscopy to provide the surgeon with a real-time measure of a tumor margin analysis or of metastatic lesions in image-guided surgery of, e.g., skin [81], breast [80] or colon [82] cancers or in brain-conserving cancer surgery [98]. Despite the many promising positive results, most of the studies are too statistically limited in the number of patients studied to take into account the variances in the population of interest, as well as the inter- and intra-observer differences. The second area of application is Raman spectroscopy and imaging for ex vivo samples, e.g., of infectious diseases, detecting pathogens in urine, blood and other body fluids, often using SERS, to design assays suitable for point-of-care applications. However, such onsite routine applications require the development of completely automated protocols for sample preparation and analysis using hand-held Raman equipment. Finally, point-of-care applications of FTIR spectroscopy and imaging, coupled with the multivariate data analysis, of biofluids, single cells and pathological (e.g., cancer) tissue sections (ex vivo spectroscopic histopathology) have seen many technological advances. However, the translation of the technology to the clinic must be driven by clinicians and pathologists. Issues like differences in tissue densities and processing artifacts can lead to erroneous results. Data analysis of the spectra and images of chemically diverse biological samples and the interpretations of the results are also challenging. The instrumental requirements for suitable clinic use, preprocessing data strategies and statistical analyses in clinical spectroscopy and data sharing protocols have been discussed [97].

Multimodal imaging can have a role in the development of all these three areas of application. For example, an integrated optical technique based on autofluorescence imaging and Raman scattering was used to analyze basal mammary ductal carcinoma and skin cell carcinoma, leading to a faster diagnoses than by histopathology and IR or Raman imaging alone [80,81]. A clinical instrument was also developed to combine Raman spectroscopy and OCT for the morphological and biochemical characterizations of skin cancer [81]. Another example is the combination of data from IR and Raman spectroscopy and the imaging of biological samples with other chemical imaging techniques, such as X-ray fluorescence (XRF), mass spectrometry imaging (MSI) and laser ablation inductively coupled plasma mass spectrometry (LA-ICP-MS). As the cell information provided by each technique is different, such as on the metallome, proteome, lipidome and metabolome, the combination of different modalities improves the definition of the cell phenotype [96].

## 5. Conclusions and Future Prospects

The various techniques that are available based on vibrational (FTIR and Raman) spectroscopy and imaging have found many and varied preclinical biomedical applications for the label-free, noninvasive in vitro studies of body fluids and cells, ex vivo tissues and biopsies and in vivo animals. Some of these are translatable to clinical medicine, depending on the development of instruments, strategies for data preprocessing, statistical analysis and sharing protocols adapted to the clinical environment [96,97]. This process can benefit from the design of new instruments, techniques and multimodal approaches to chemical probes. Raman tweezers able to obtain Raman spectra of optically trapped biological cells are being used in the acquisition of Raman images [99]. A very recent and promising technique is micro-photoacoustic infrared spectroscopy (microPAS), allowing photoacoustic (PA) IR spectroscopy measurements of small samples using conventional FTIR spectrometers, providing microscopic images with spatial resolutions in the 20–100-μm range [100]. In relation to chemical probes, the super-resolution imaging of SERS tag hot spots provides spatial resolutions on the order of 1–10 nm [101]. The multiplexing capabilities of SERS tags can be used in many imaging applications, such as sensing of the cellular environment, multifunctional imaging and theragnostic platforms [102]. The combination of various imaging modalities using the same SERS-based nanoplatform has often included modalities, such as fluorescence imaging or PAI, with similar characteristics—high sensitivity and spatial resolution, limited tissue penetration and a small field of view. The limitations of SERS-based Raman imaging can be best compensated by adding functionalities active in complementary modalities, such as MRI/CT/US. Thus, the careful selection of different functionalities on a single SERS-based platform can facilitate progress towards specialized and personal medical procedures.

## Figures and Tables

**Table 1:** Main Fourier-transform infrared (FTIR) spectral regions and group vibrations present in the biological macromolecules.

Spectral Region, λ (cm^−1^)	Group Vibrations	Main Contributing Macromolecules
3600–3050	OH stretch	carbohydrates
3050–2800	CH, CH_2_, CH_3_ stretch	fatty acids, proteins
3100–2550	SH, NH stretch	proteins
1800–1700	C=O stretch	lipid esters
1700–1500	Amide I/II	proteins
1443–1391	CH_2_, CH_3_ bending	fatty acids, proteins, nucleic acids
1340–1155	Amide III, CH_2_ wagging	collagen
1240–1080	Phosphate stretch	nucleic acids, phospholipids
1200–900	C-O, C-C stretch	glycogen, carbohydrates
1450–600	Carbon skeleton fingerprint	all
800–100	CCO deformation	all

**Table 2:** Comparison of the features of IR/Raman imaging with those of the other main modalities used in research and clinics. These features depend on the experimental configuration [2,66]. PET: Positron Emission Tomography, SPECT: Single-Photon Emission-Computed Tomography, SERS: Surface-Enhanced Raman Scattering, SORS: Spatially Offset Raman Spectroscopy and NIRF: Near Infrared Fluorescence.

Imaging Modality	Spatial Resolution	Temporal Resolution	Penetration Depth	Sensitivity
IR	2.5–6 μm	s/min	10 μm–1 mm	10^−4^–10^−5^ M
Raman	0.5–1 μm 20–100 nm (SERS)	s/min	200–300 μm 20–100 μm (SERS) ~1 mm (SORS)	10^−6^–10^−7^ M 10^−12^–10^−15^ M (SERS)
MRI	25–100 μm (preclinical) ~1 mm (clinical)	s/h	unlimited	10^−3^–10^−5^ M
Luminescence	2 to 3 mm 1–10 μm (NIRF)	s/min	<2 cm	10^−9^–10^−12^ M
Intravital microscopy	100–300 nm	ms/s	~1 mm	10^−15^–10^−17^ M
Resonance energy transfer	2 to 3 mm	s	<2 cm	10^−6^–10^−10^ M
Optical coherence tomography (OTC)	1 μm	s	~2 to 3 mm	10^−10^–10^−11^ M
Photoacoustic imaging (PAI/PAT)	5 μm–1 mm (depth-dependent)	s/min	<6 cm	10^−9^–10^−11^ M
Ultrasound imaging (US)	10–100 μm (at ~mm depth); 1 to 2 cm (at ~cm depth)	s/min	1 cm	10^−6^–10^−9^ M
γ-imaging	1–10 mm	min	unlimited	10^−10^–10^−11^ M
PET	<1 mm (preclinical), ~5 mm (clinical)	s/min	unlimited	10^−11^–10^−12^ M
SPECT	0.5–2 mm (preclinical), 8–10 mm (clinical)	min	unlimited	10^−10^–10^−11^ M
Computed tomography (CT)	25–200 μm (preclinical), 0.5–1 mm (clinical)	s/min	unlimited	10^−3^ M
